# Dental Implants – Perceiving Patients’ Satisfaction in Relation to Clinical and Electromyography Study on Implant Patients

**DOI:** 10.1371/journal.pone.0140438

**Published:** 2015-10-14

**Authors:** Mohammad Khursheed Alam, Shaifulizan Abdul Rahaman, Rehana Basri, Tiffany Tang Sing Yi, Justin Wong Si-Jie, Soumendra Saha

**Affiliations:** 1 Orthodontic Unit, School of Dental Science, Universiti Sains Malaysia, Kota Bharu, Kelantan, Malaysia; 2 Oral and Maxillofacial Surgery Department, School of Dental Science, Universiti Sains Malaysia, Kota Bharu, Kelantan, Malaysia; 3 Craniofacial Biology, School of Dental Science, Universiti Sains Malaysia, Kota Bharu, Kelantan, Malaysia; 4 Department of Exercise and Sports Science, School of Health Science, Universiti Sains Malaysia, Kota Bharu, Kelantan, Malaysia; University of Brescia, ITALY

## Abstract

The aim of this study is to evaluate the satisfaction of patients with posterior implants in relation to the clinical success criteria and surface electromyography (sEMG) findings of the masseter and temporalis muscles. Total 42 subjects were investigated. Twenty one subjects with posterior dental implants were interviewed using a questionnaire and the clinical success criteria were determined based on The International Congress of Oral Implantologists. The myofunction of the masticatory muscles were assessed using sEMG (21 subjects) and compared to the control group of subjects without implants (21 subjects). Out of 21 subjects, all were satisfied with the aesthetics of their implant. Twenty of them (95.2%) were satisfied with its function and stability. As for clinical criteria, 100% (50) of the implants were successful with no pain, mobility or exudates. sEMG findings showed that patients have significantly lower (p<0.01) basal or resting median power frequency but with muscle burst. During chewing, control subjects showed faster chewing action. There was no difference in reaction and recovery time of clenching for both groups. In conclusion, the satisfaction of implant patients was high, and which was in relation to the successful clinical success criteria and sEMG findings.

## Introduction

A dental implant is an artificial tooth root that is placed into the jaw to replace a tooth or teeth lost due to periodontal disease, tumors, infections, trauma or developmental anomalies [1]. Dental implant is known to be one of the most preferred treatment options as they are the nearest equivalent replacement to the natural tooth.

Proper assessment and follow up of the implant must be carried out to determine the effect of the implant on function of masticatory muscles. Clinical assessment is important to establish success criteria for implants [1]. Currently, clinical examination methods such as those used in periodontology have been widely applied in implant dentistry [2]. The clinical success criteria are adapted from The International Congress of Oral Implantologists (ICOI) Pisa Consensus Conference to determine the clinical success of the implant.

Surface electromyography (sEMG) is a non-invasive index used to identify muscular activity of the face (masseter) and head (temporalis) muscles directly [3,4]. sEMG is performed using an instrument called electromyography, to produce a record called an electromyogram. Generally, the masseter and the temporalis are synergists and function concurrently. While the temporalis provides a basis for mandibular balance and postural control, the masseter is used during grinding and chewing [5].

However, no research in Malaysia has been done regarding surface electromyography (sEMG) results of patients with dental implants. This research is to evaluate the satisfaction of patients with posterior implants in relation to the clinical success criteria and the surface electromyography (sEMG) findings of the masseter and temporalis muscles. As far as search of literature and current knowledge is concerned, no prior surface electromyography data exists of dental implant patients; therefore this would be a pilot finding.

### Specific objectives

1. To determine the satisfaction and clinical success criteria of patients with posterior implants.
2. To assess myofunction of face (masseter) and head (temporalis) muscles of patients with posterior implants and control group by surface electromyography (sEMG).
3. To compare the satisfactionof patients with posterior implants with their clinical success criteria and myofunctional effect of masseter and temporalis muscles.

### Hypotheses

H0: Patients who are satisfied with their functioning posterior implant fall under the success clinical criteria. There is no significant difference between the electromyography studies of posterior implant patients and control group. Patients who are satisfied with their functioning implant have similar masseter and temporalis myofunction compared to control group.

## Materials and Methods

All subjects were informed verbally and as well as written consent were under taken. Ethical approval was undertaken by the Universiti Sains Malaysia human ethics committee. Letter from Human Research Ethics Committee (HREC) has assigned a study protocol code USM/JEPeM/1405200.

This is a pilot cross-sectional and case control study to evaluate the satisfaction of patients with posterior implants in relation to their clinical and surface electromyography findings. The dental implants were placed within the year 2011 to 2013. Data were collected using questionnaire, clinical success criteria and muscle activity signals taken by the sEMG (ProComp Infiniti Encoder SA7500, Montreal, Quebec, Canada) machine and stored as files. These files were then analyzed using the machines accompanying computer software.

The sampling frame consisted of patients who have either single or multiple dental implants in the posterior region (1^st^ or 2^nd^ molar [only] of either maxilla or mandible) with opposing natural dentition. All patients underwent standard aseptic surgical protocol for implant placements under local anesthesia by a specialist Oral and Maxillofacial Surgeon. In term of complexity in implant surgery, all the patients has similar grade of difficulty which were straight forward delayed type of placement that represents the complete healing of hard and soft tissue. Complete instructions of the procedures and information sheet for the research were given to the patients. Twenty one subjects (7 males and 14 females) with functional posterior dental implants were part of this study. All subjects received similar type of implants (Implantium Implants System, Dentium, Seoul, Korea). The same procedure and protocols was carried out on all subjects by the same operator.

### Inclusion criteria

The subjects chosen fulfilled the following inclusion criteria -

- Patients who received posterior dental implants in HUSM within 2011 to 2013
- Consented for this study
- Patients were 18 years old and above

### Exclusion criteria

- Patients who received anterior dental implants in HUSM
- Patients with physical handicaps that would interfere with the ability to perform adequate oral hygiene

### Sample size calculation

**Fig 1:**
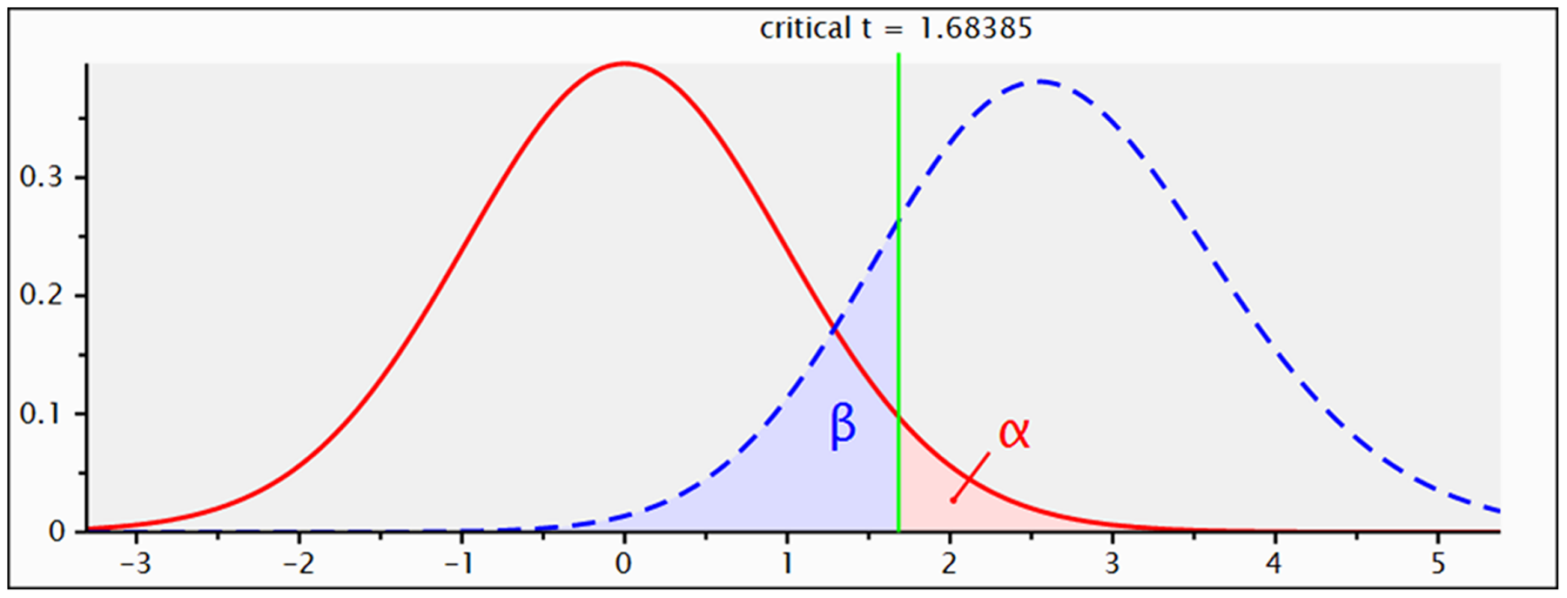
Graphical presentation of sample size calculation.

Central & Non-Central Distribution (Fig 1)

**t tests—Means:** Difference between two independent means (two groups)

**Analysis:** A priori: Compute required sample size

**Input:**  Tail(s)        = One

****Effect size d      = 0.8

****α err prob       = 0.05

****Power (1-β err prob)  = 0.80

****Allocation ratio N2/N1 = 1

**Output:** Noncentrality parameter δ = 2.592296

****Critical t       = 1.683851

****Df          = 40

****Sample size group 1  = 21

****Sample size group 2  = 21

****Total sample size    = 42

****Actual power     = 0.816788

For this experiment, 42 adult participants (21Control & 21Participants from surgical implant patient population) will be selected using G power 3.1.9 software [6]. The power of the study was set at 0.80 with 95% confidence interval and the effect size was set at 0.8. Hence, the total sample size for this research is 42 subjects.

All subjects were given a questionnaire that contains questions regarding their perioperative experience undergoing the implant treatment in relation to all phases of treatment. The questionnaire consists of 7 parts which are demographic background, source of information, reason for undergoing implant treatment, discomfort related to all phases of treatment, functional and aesthetic satisfaction, period of implant placement and number of dental visits after implant placement. Patients who did not understand the questions were explained verbally in the simplest term for their better understanding about the question. The subjects were then examined clinically on a dental chair to determine the number of implants, the location of the implant and clinical criteria of the dental implant. The clinical criteria were divided into 4 categories, which are success, satisfactory survival, compromised survival and failure. The implant is then categorized accordingly after thorough examination of surrounding tissues of the implant, pain, mobility and pus discharge. The instruments used for the clinical examination were an examination tray, an explorer, a periodontal probe and tweezers. After the clinical examination, surface electromyography (sEMG) of the masseter and temporalis muscles was done using an electromyograph device. The patients were given clear instructions before the sEMG recording started.

The instructions given were:

- To keep the head and body still as movement of the head and neck region or the body will affect the sEMG results.
- Do not move the tongue as it will result in stimulation to the muscles thus affecting the results.
- A chewing gum will be given but chewing will only start on signal provided.
- The 3 surface leads will be placed at 4 areas: left masseter, right masseter, left temporal region, right temporal region.
- The sEMG recording will start at the left masseter after surface lead placement with the patients’ masticatory muscles being at rest for 10 seconds, followed by 10 seconds of chewing on the left side, then 10 seconds of chewing on the right side, followed by 10 seconds of rest and 10 seconds of continuous even clenching, ending with another 10 seconds of rest.
- Next, the surface leads will be placed at the right masseter region for sEMG recording with the same instructions.
- For the left and right temporalis muscle, patients are instructed to rest for 10 seconds, followed by continuous even clenching for 10 seconds and ending with another 10 seconds of rest.

### Statistical analysis

The data were verified and analysed statistically using IBM SPSS Statistics Version 22.0 (Armonk, NY: IBM Corp.) with confidence level set at 5% (P < 0.05) to test for significance. Mean amplitude of the muscles (masseter and temporalis) between male and female patients and two age groups were analyzed by t test. Mean amplitude of the muscles (masseter and temporalis) between patients and the control group during basal stage (resting), post chew resting stage, post clenching basal stage (resting), left chewing stage, right chewing stage and during clenching stage was analyzed by t test while One-Way ANOVA was done to investigate mean amplitude of the muscles (masseter and temporalis) between patients with one sided implants, patients with two sided implants and the control group without implants.

**Fig 2:**
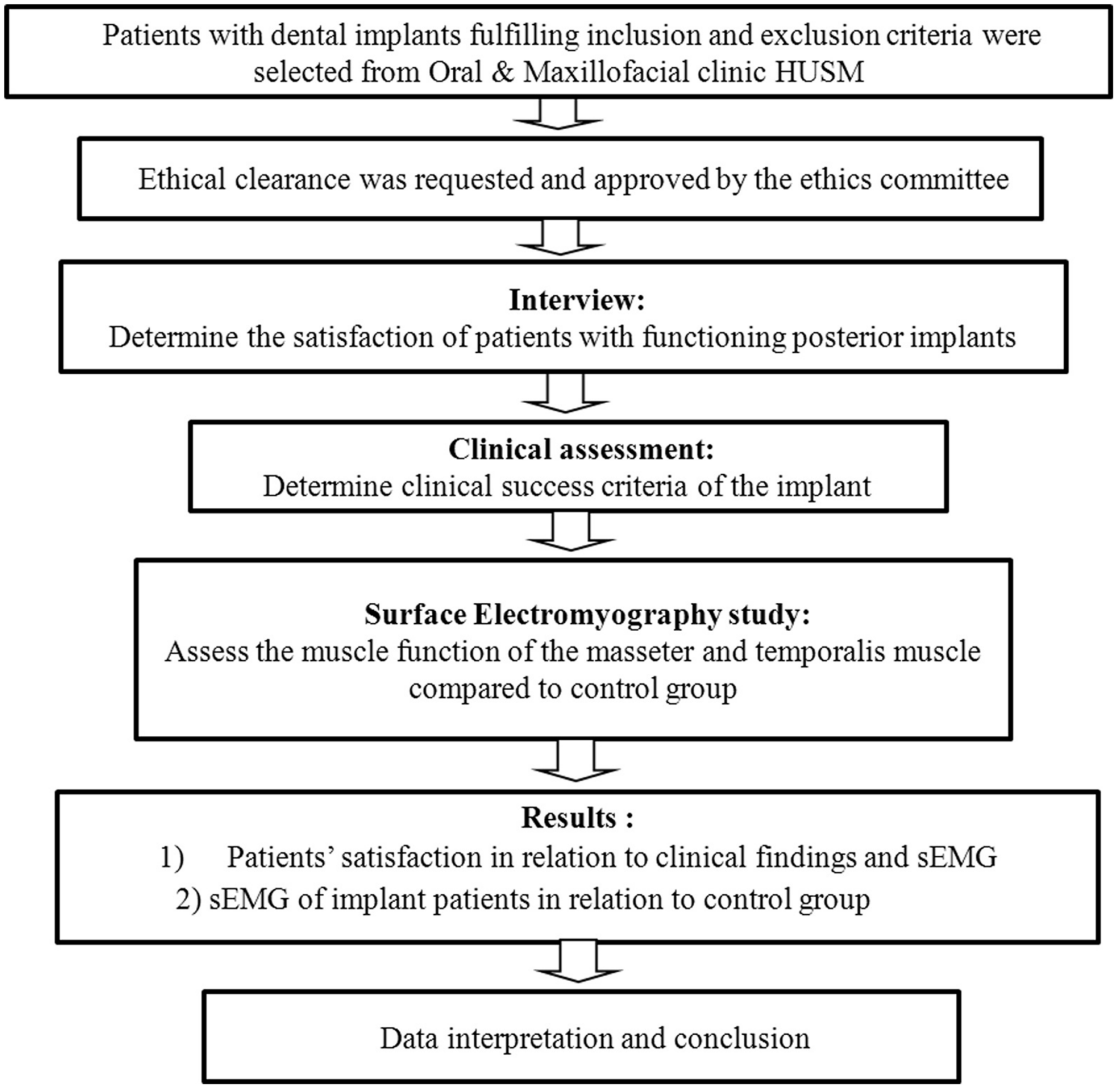
Flow Chart of the Study.

Flow Chart of the Study is shown in Fig 2.

## Results

### Demographic data

Table 1 shows the demographic data of the patients. The age of the patients ranges from 31–65. Employment status shows that 38.1% (8) of the patients were unemployed, mainly consisting of housewives and retirees.

**Table 1:** Demographic data of the patients.

Variable	Frequency	Percentage (%)
Age group		
31–40 [50<]	6	28.6
41–50 [50<]	8	38.1
51–60 [50>]	6	28.6
61–70 [50>]	1	4.8
Mean age = 47.95 (SD 9.222)		
Gender		
Male	7	33.3
Female	14	66.7
Employment status		
Unemployed	8	38.1
Government	11	52.4
Private	2	9.5
Marital status		
Single	3	14.3
Married	18	85.7
Divorced	0	0.0
Educational level		
None	0	0.0
Primary	0	0.0
Secondary	7	33.3
University	14	66.7

### Source of information

Table 2 shows the source of information regarding implants of the patients.

**Table 2:** Source of information of implants of the patients.

Source of Information	Frequency	Percentage (%)
Referring dentists	3	14.3
Relatives and friends	13	61.9
Television, radio or internet	5	23.8
Newspapers	0	0.0
Medical doctors	0	0.0

### Reason for undergoing implant treatment

Table 3 shows the reason for undergoing implant treatment.

**Table 3:** Reason of undergoing implant treatment.

Reason	Frequency	Percentage (%)
To restore lost teeth	14	66.7
To relieve discomfort from previous appliance	7	33.3
To improve the stability of a removable denture	0	0.0
Eating habits	0	0.0
Esthetics	0	0.0

### Discomfort related to all phases of treatment

Eighteen of the patients (85.7%) denied of any discomfort while 3 (14.3%) of them finds discomfort due to the procedure taking a long time. Twenty of the patients (95.2%) did not find the implant treatment to be traumatic while only 1 of them (4.8%) feels that implant treatment is traumatic. Fourteen of the patients (66.7%) did not experience any swellings throughout the implant treatment while 7 of them (33.3%) experienced swelling during treatment. Out of the 7 patients, four of them had mild swelling, 2 had moderate swelling while 1 experienced severe swelling. Twelve of the patients (57.1%) did not experience any pain throughout the implant treatment while 9 of them (42.9%) experienced pain during treatment. Twenty of the patients (95.2%) did not experience any post-operative discomfort other than pain or swelling while 1 of them (4.8%) experienced numbness post operation.

### Functional & aesthetical satisfaction

Twenty of the patients (95.2%) were satisfied with the function of their dental implants while 1 of them (4.8%) was not satisfied with its function. Regarding aesthetics, all of the patients (100%) were satisfied with the appearance of their dental implants. Eighteen of the patients (85.7%) felt that the dental implant was a part of them while 3 of them (14.3%) felt that the implant was strange. Patients were asked to rate the stability of their implant into poor, moderate or good. Twenty of the patients (95.2%) felt that the stability of their dental implants were good while 1 of them (4.8%) rated it moderate. After the placement of implant, 16 of the patients (76.2%) went for 1 dental visit, 2 of them (9.5%) came for 2 visits, another 2 (9.5%) came for 3 visits while 1 (4.8%) went for 4 visits.

### Clinical examination of the implants

The total number of implants examined was 55. Table 4 shows that patients were presented with 1 up to 6 implants. 47.3% of the implants were located at the left side of the dentition, while 52.7% were located at the right side.

**Table 4:** Total number of implants examined.

Number of implants	Frequency	Percentage (%)
1	4	19.0
2	9	42.9
3	3	14.3
4	3	14.3
5	0	0.0
6	2	9.5

Table 5 shows that all the 55 implants examined (100%) fell into the successful clinical criteria with no pain or tenderness upon function, no mobility and no exudates history.

**Table 5:** Clinical success criteria of the implants.

Clinical success criteria	Frequency	Percentage (%)
Success	55	100.0
Satisfactory survival	0	0.0
Compromised survival	0	0.0
Failure	0	0.0

### sEMG results

**Fig 3:**
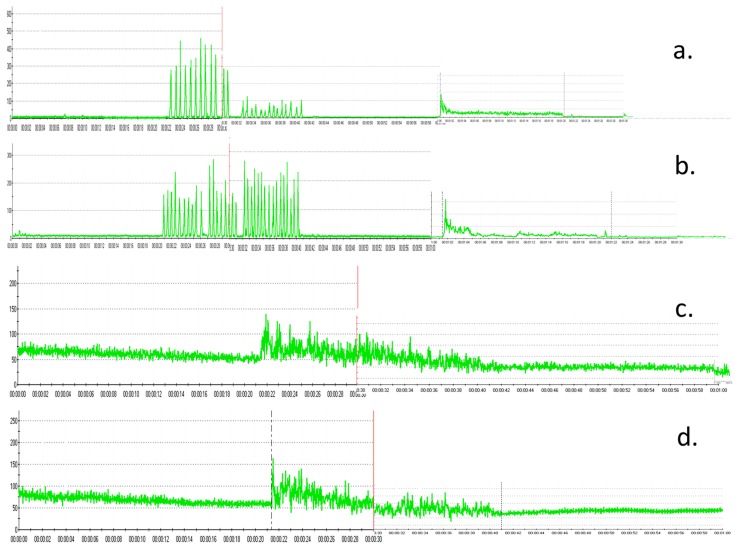
Representative sEMG amplitude in the control group. a. left masseter muscle b. right masseter muscle c. left temporalis muscle d. right temporalis muscle.

Fig 3 shows the representative sEMG amplitude of the left masseter muscle (Fig 3a), right masseter muscle (Fig 3b), left temporalis muscle (Fig 3c) and right temporalis muscle (Fig 3d) in the control group.

**Fig 4:**
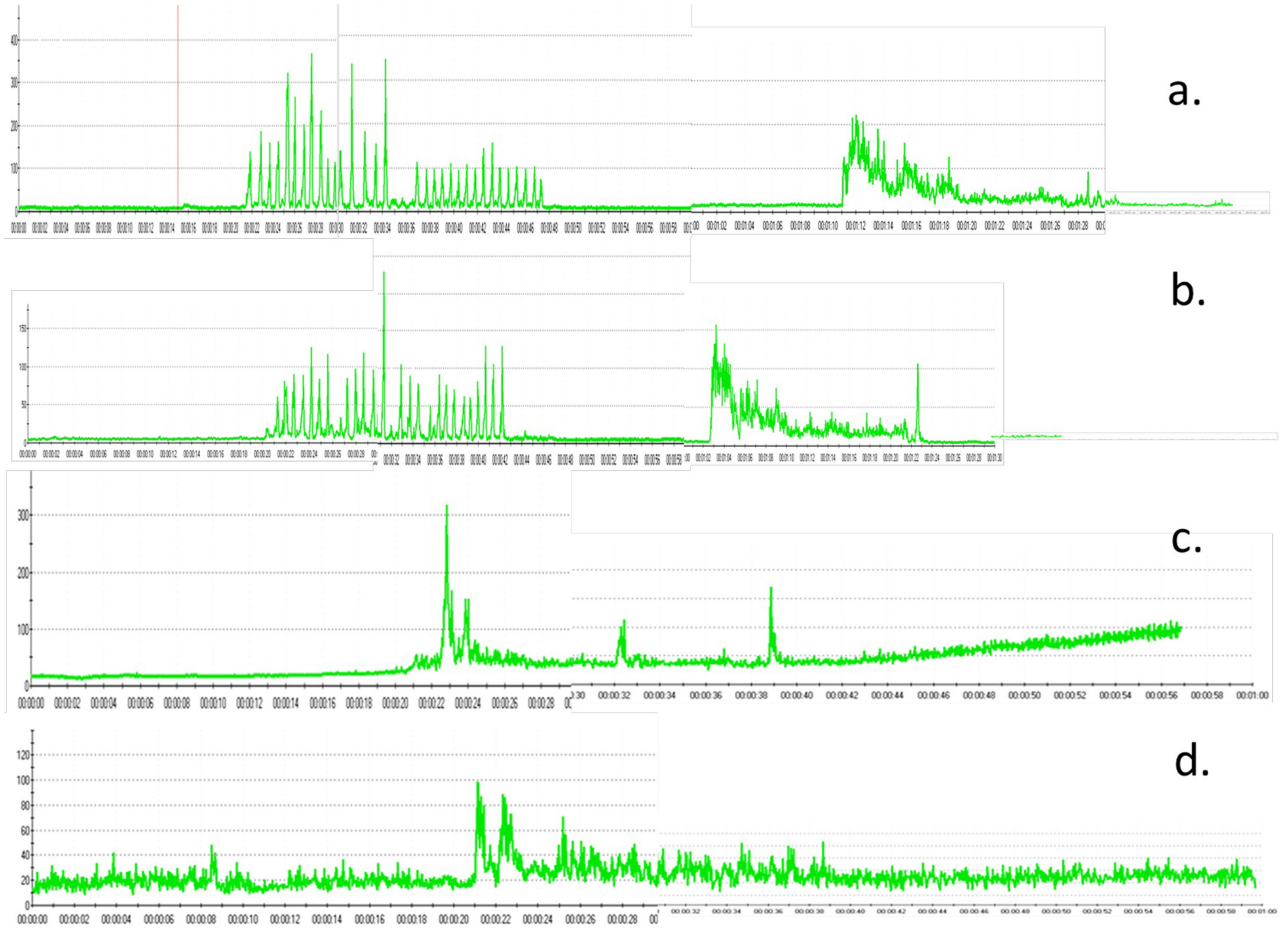
Representative sEMG amplitude in the patient group. a. left masseter muscle b. right masseter muscle c. left temporalis muscle d. right temporalis muscle

Fig 4 shows the representative sEMG amplitude of the left masseter muscle (Fig 4a), right masseter muscle (Fig 4b), left temporalis muscle (Fig 4c) and right temporalis muscle (Fig 4d) in the patients group.

**Fig 5:**
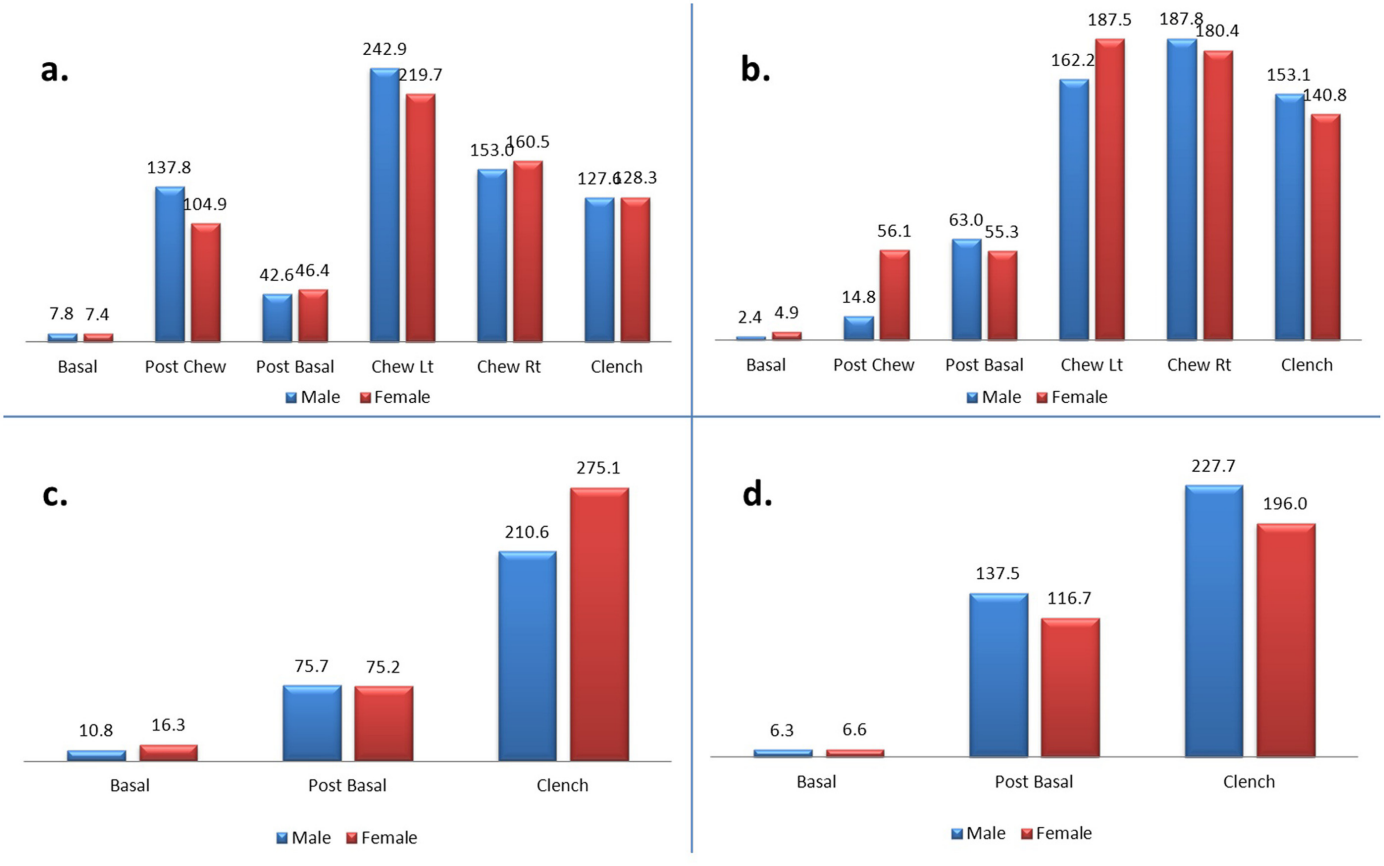
a-d. Mean amplitude of the left masseter (a), right masseter (b), left temporalis (c) and right temporalis (d) muscles between male and female patients.

Fig 5a–5d shows the mean amplitude of the left masseter (a), right masseter (b), left temporalis (c) and right temporalis (d) muscles between male and female patients. No significant differences were observed in all stages when compared between male and female patients.

**Fig 6:**
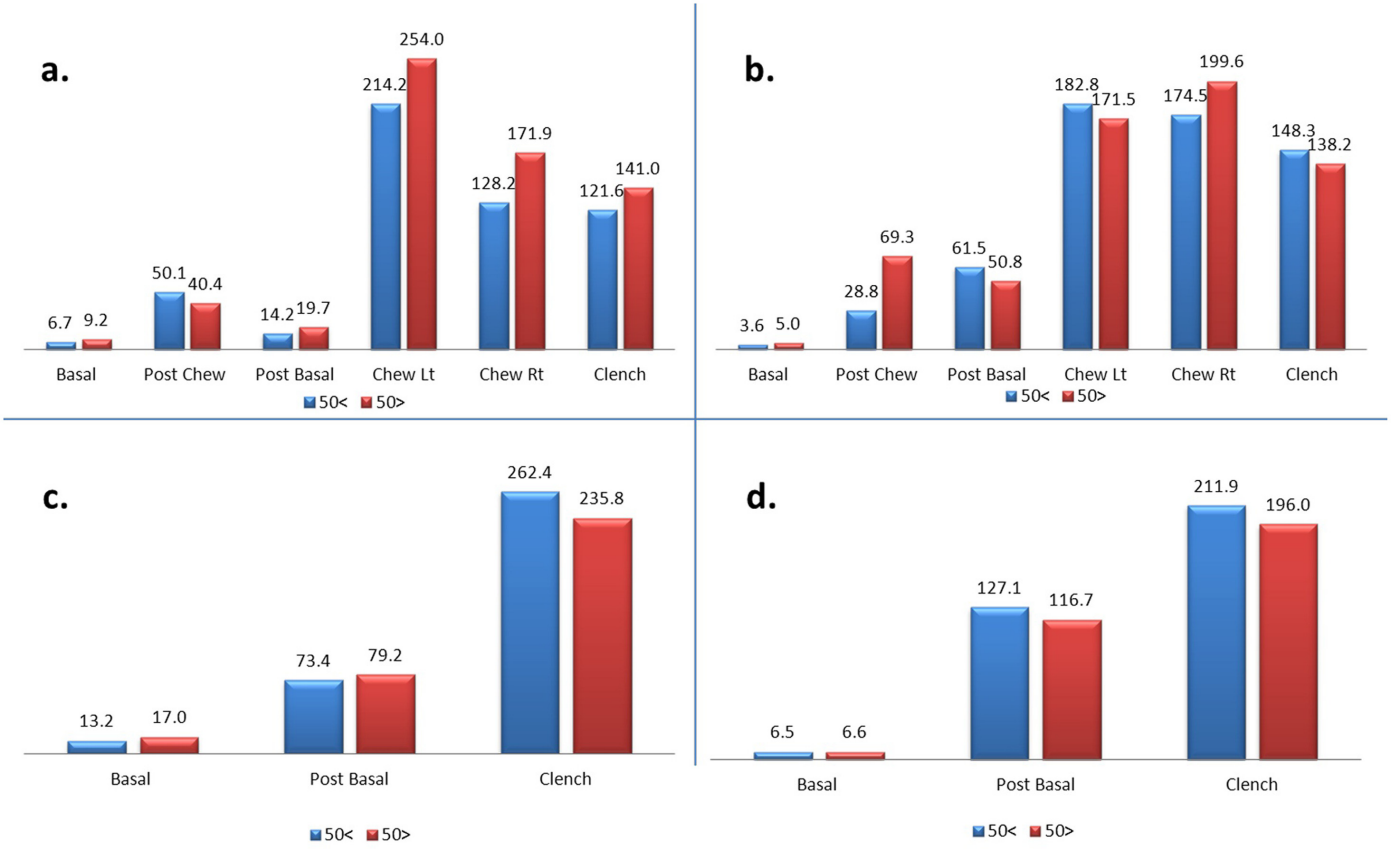
a-d. Mean amplitude of the left masseter (a), right masseter (b), left temporalis (c) and right temporalis (d) muscles between two age groups (50< vs 50>) of the patients.

Fig 6a–6d shows the mean amplitude of the left masseter (a), right masseter (b), left temporalis (c) and right temporalis (d) muscles between two age groups (50< vs 50>) of the patients. No significant differences were observed in all stages when compared between two age groups of the patients.

**Fig 7:**
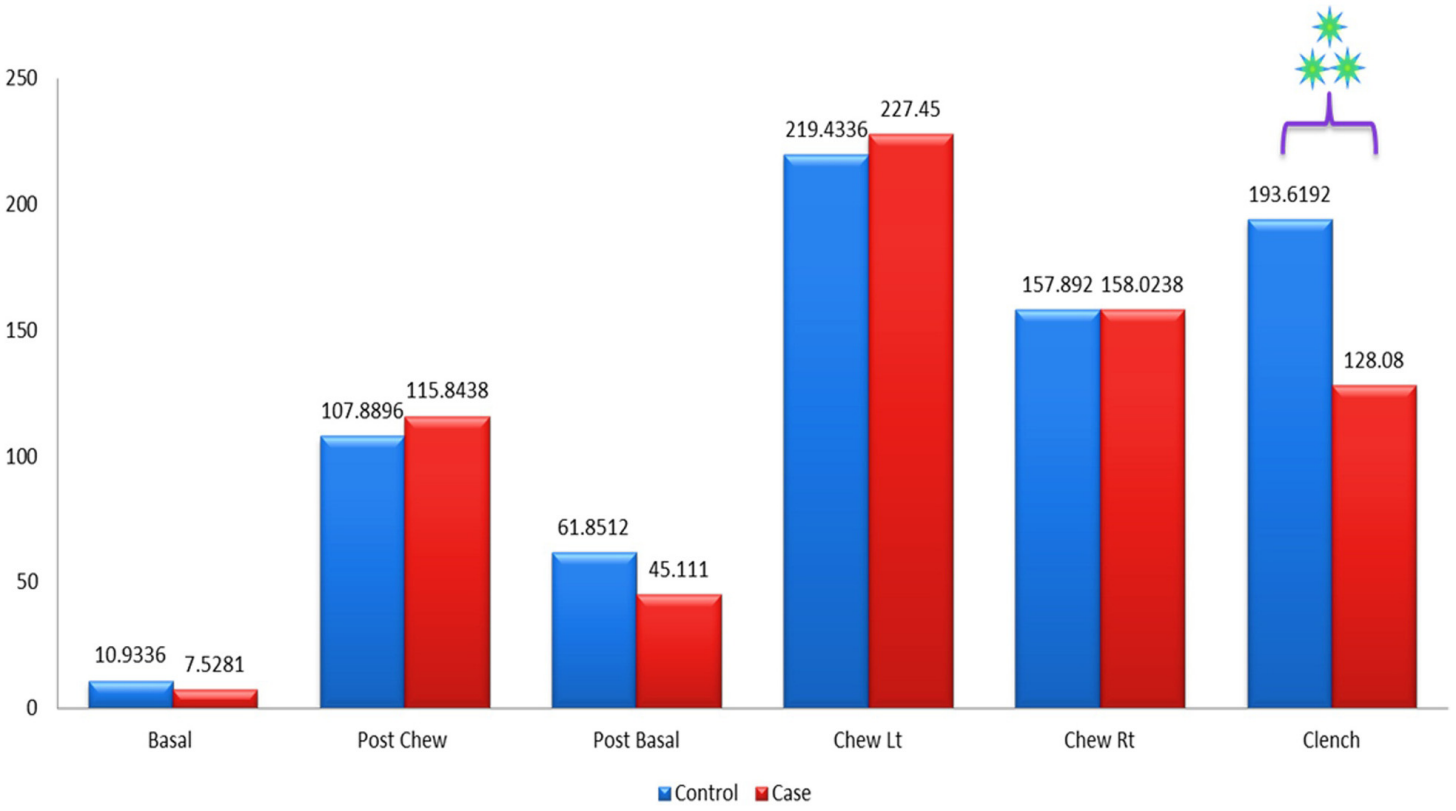
Mean amplitude of the left masseter muscle between control and patient.

Fig 7 shows the mean amplitude of the left masseter muscles between patients and the control group during basal stage (resting), post chew resting stage, post clenching basal stage (resting), left chewing stage, right chewing stage and during clenching stage. The mean amplitude of the left masseter muscles was not significant in all stages when compared between patients and control group except for the clenching stage (p<0.001). The mean amplitude of the control group was 193.62mV while the mean amplitude of the patients was 128.08mV.

**Fig 8:**
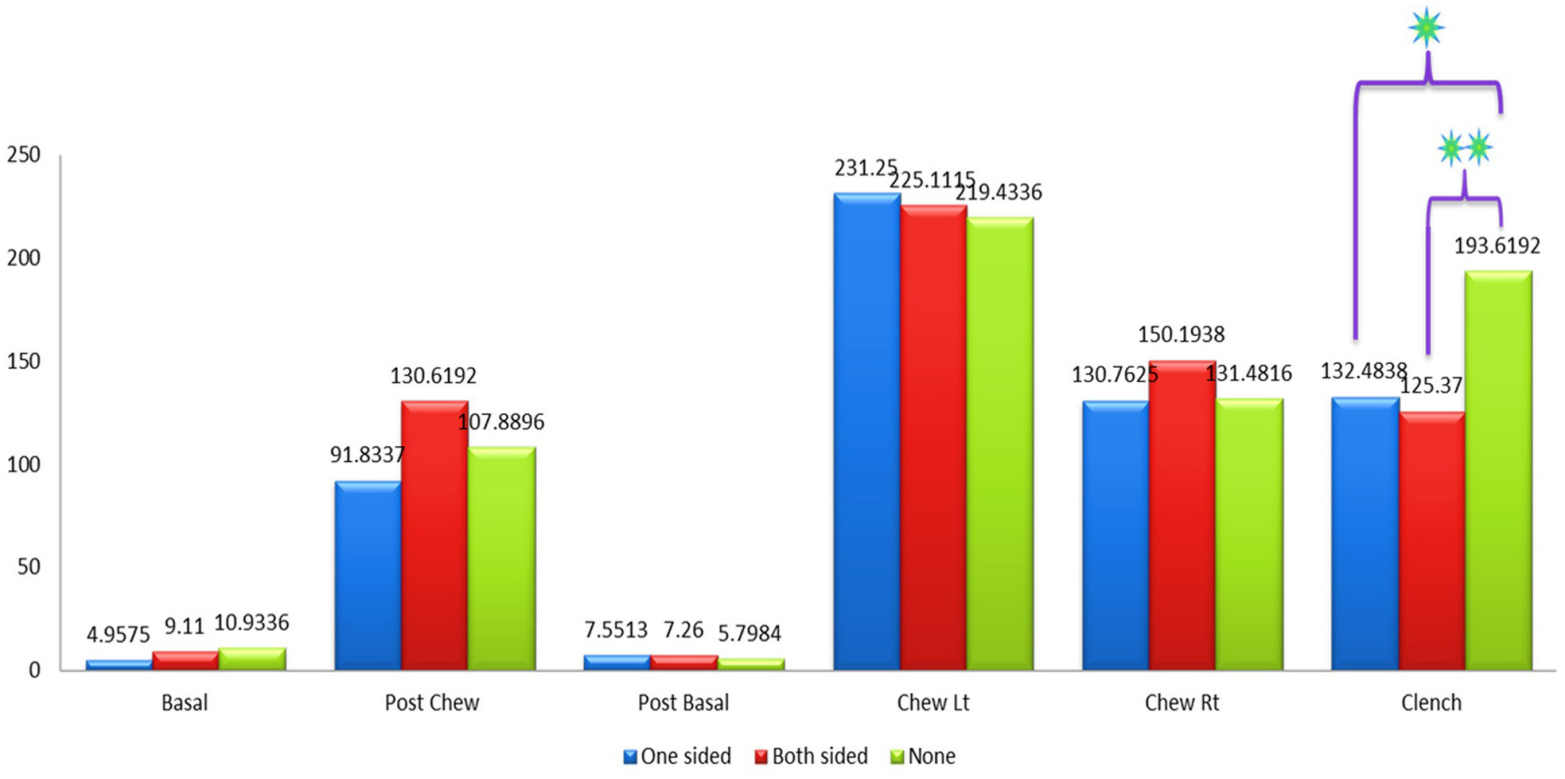
Mean amplitude of the left masseter muscle between control and patient based on the location of implant.

Fig 8 shows the mean amplitude of the left masseter muscles between patients with one sided implants, patients with two sided implants and the control group without implants during basal stage (resting), post chew resting stage, post clenching basal stage (resting), left chewing stage, right chewing stage and during clenching stage. The results were not significant in all states except for the clenching stage when compared between patients with one sided implants and the control group (p<0.05) while comparison between patients with two sided implants and the control group also showed significant difference in their results (p<0.01).

**Fig 9:**
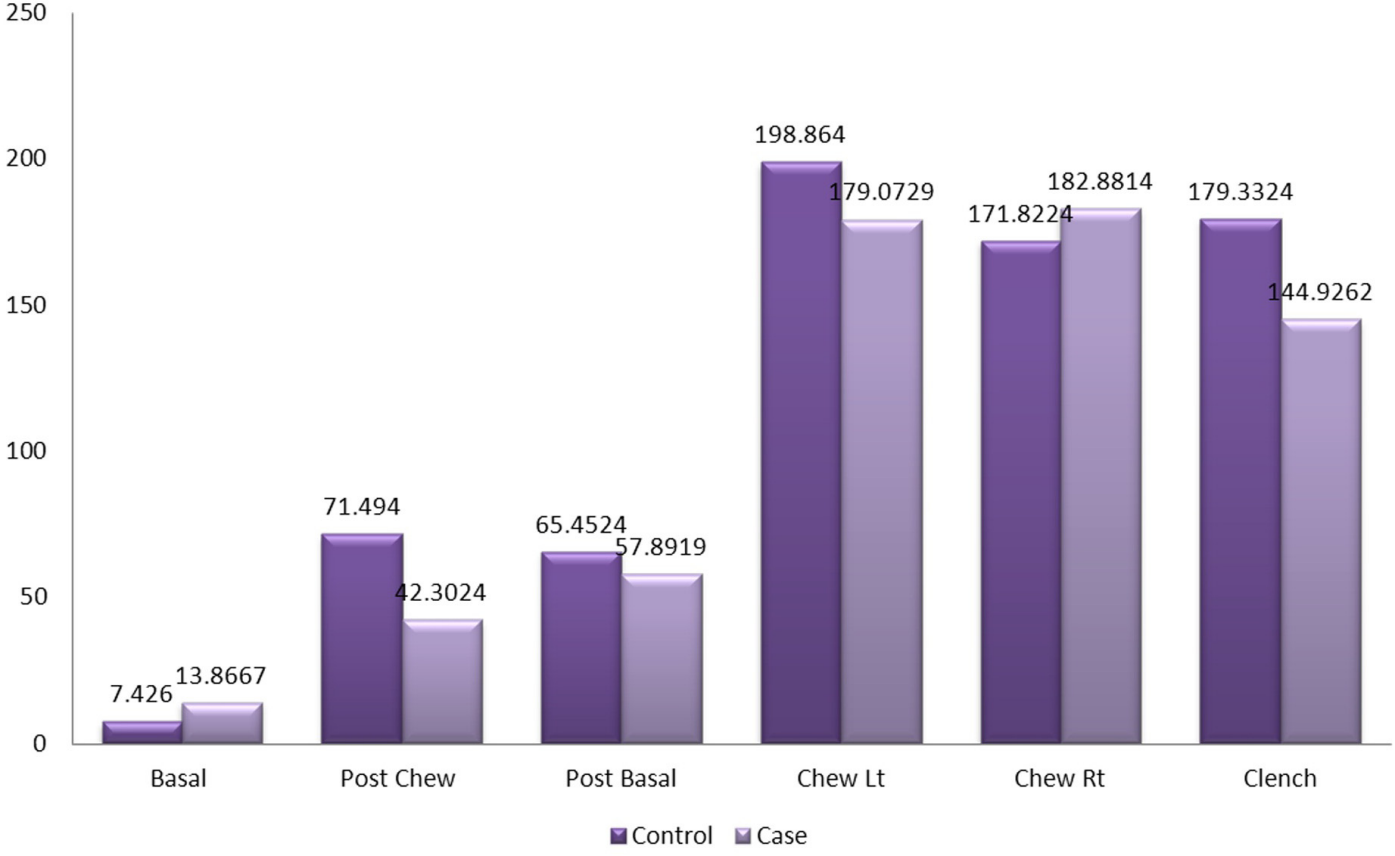
Mean amplitude of the right masseter muscle between control and patient.

Fig 9 shows that the mean amplitude of the right masseter muscles between patients and the control group during basal stage (resting), post chew resting stage, post clenching basal stage (resting), left chewing stage, right chewing stage and during clenching stage were not significant.

**Fig 10:**
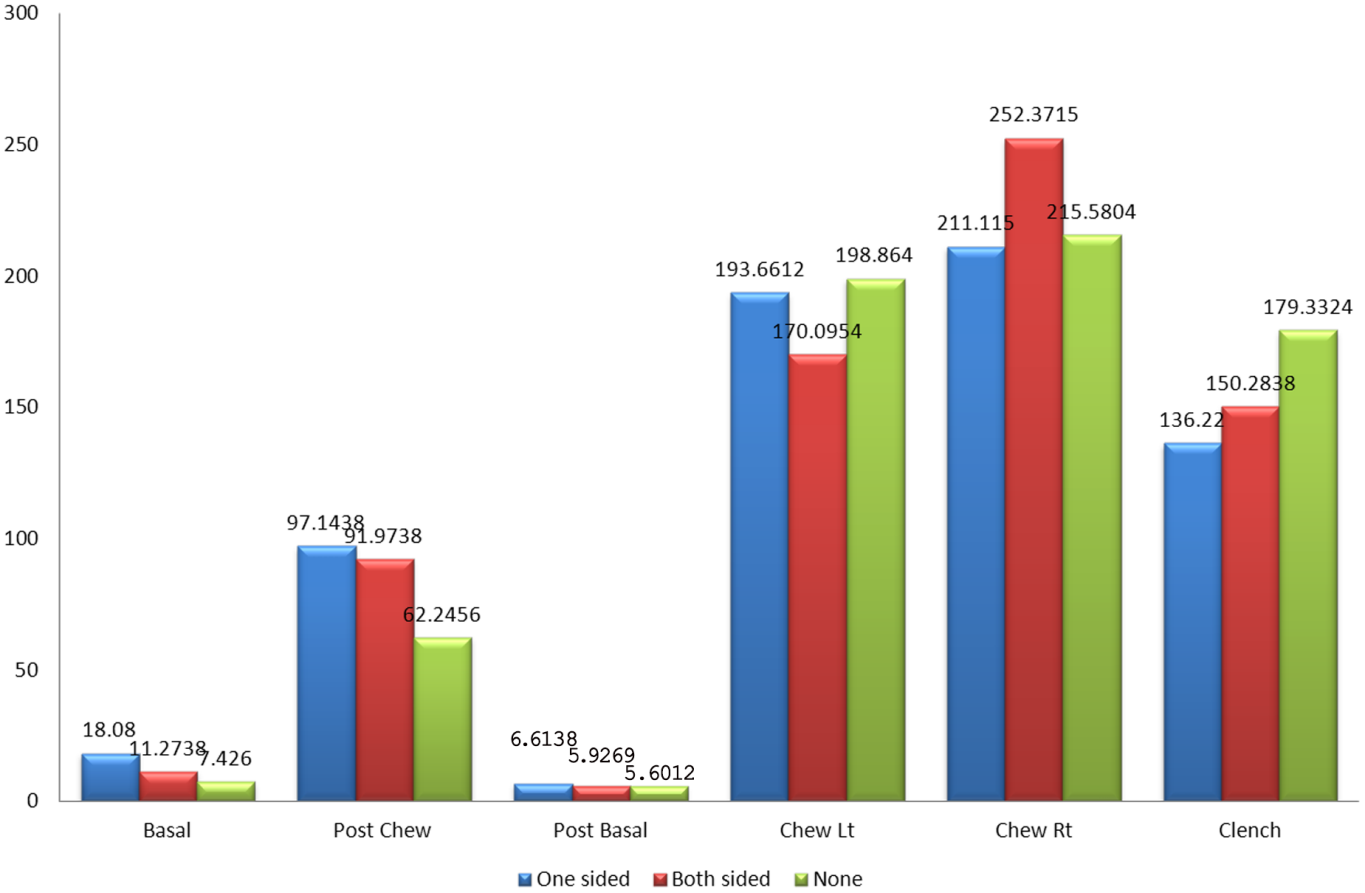
Mean amplitude of the right masseter muscle between control and patient based on the location of implant.

Fig 10 shows that the mean amplitude of the right masseter muscles between patients with one sided implants, patients with two sided implants and the control group without implants during basal stage (resting), post chew resting stage, post clenching basal stage (resting), left chewing stage, right chewing stage and during clenching stage were not significant as well.

**Fig 11:**
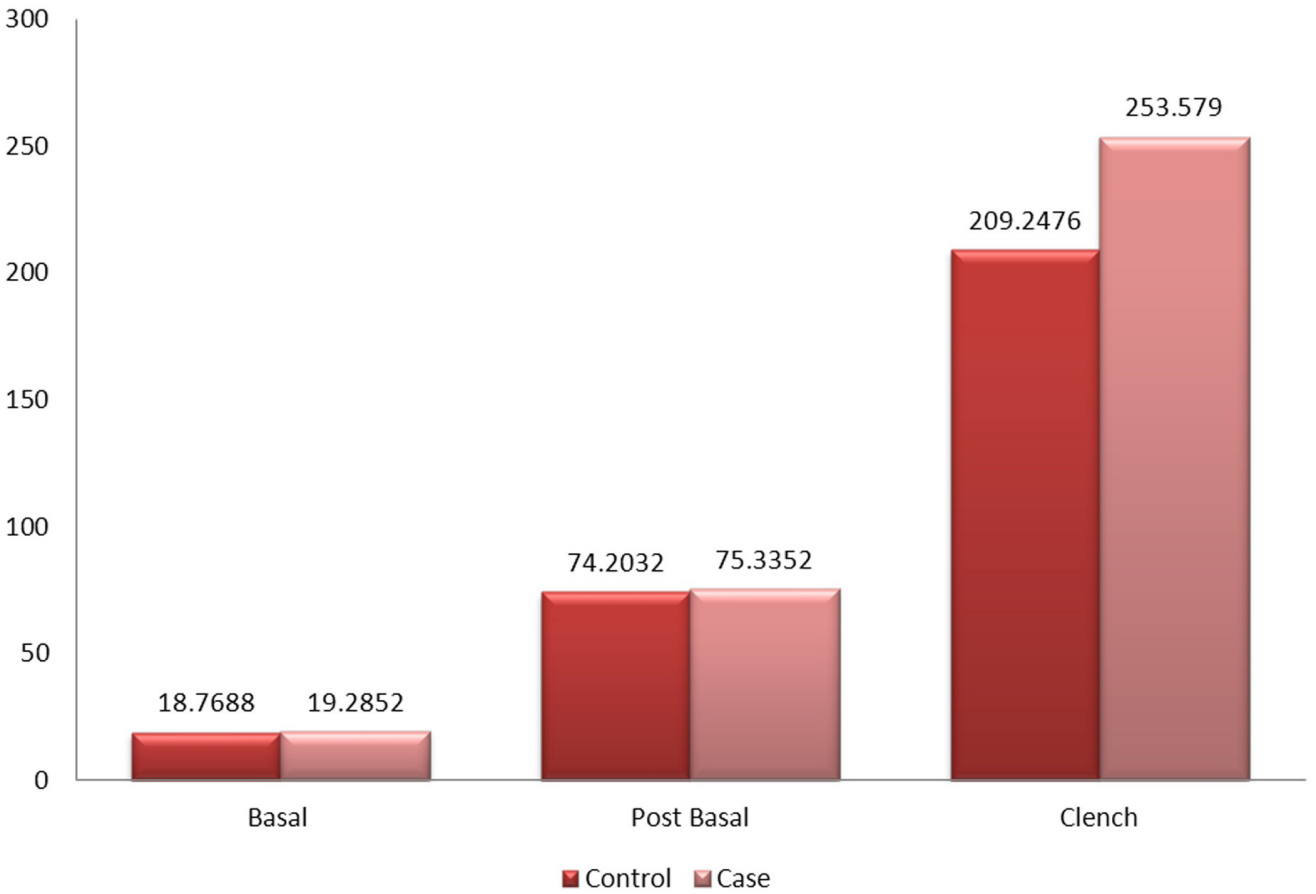
Mean amplitude of the left temporalis muscle between control and patient.

Fig 11 shows that the mean amplitude of the left temporalis muscles between patients and the control group during basal stage (resting), post chew resting stage, post clenching basal stage (resting), left chewing stage, right chewing stage and during clenching stage were not significant.

**Fig 12:**
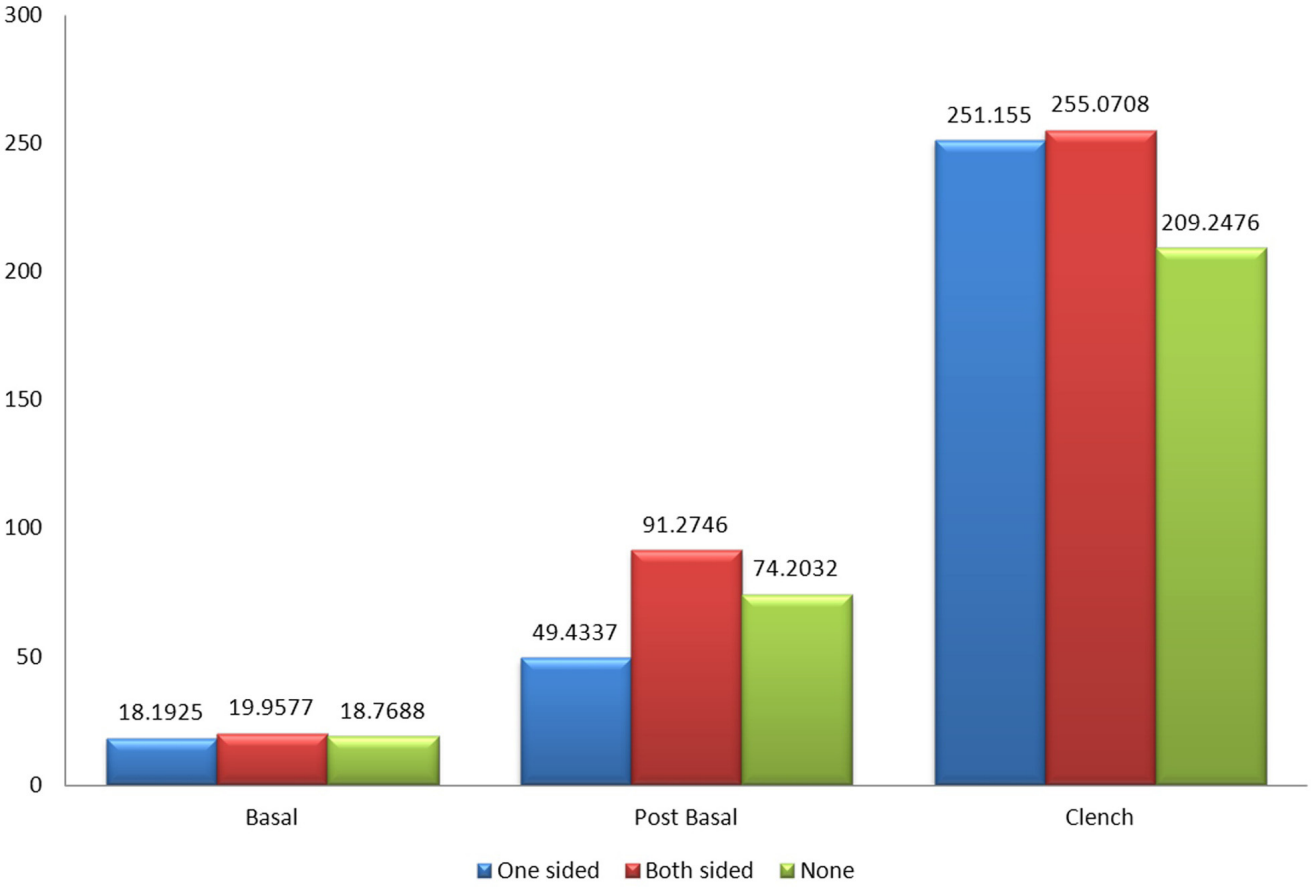
Mean amplitude of the left temporalis muscle between control and patient based on the location of implant.

Fig 12 shows that the mean amplitude of the left temporalis muscles between patients with one sided implants, patients with two sided implants and the control group without implants during basal stage (resting), post chew resting stage, post clenching basal stage (resting), left chewing stage, right chewing stage and during clenching stage were not significant as well.

**Fig 13:**
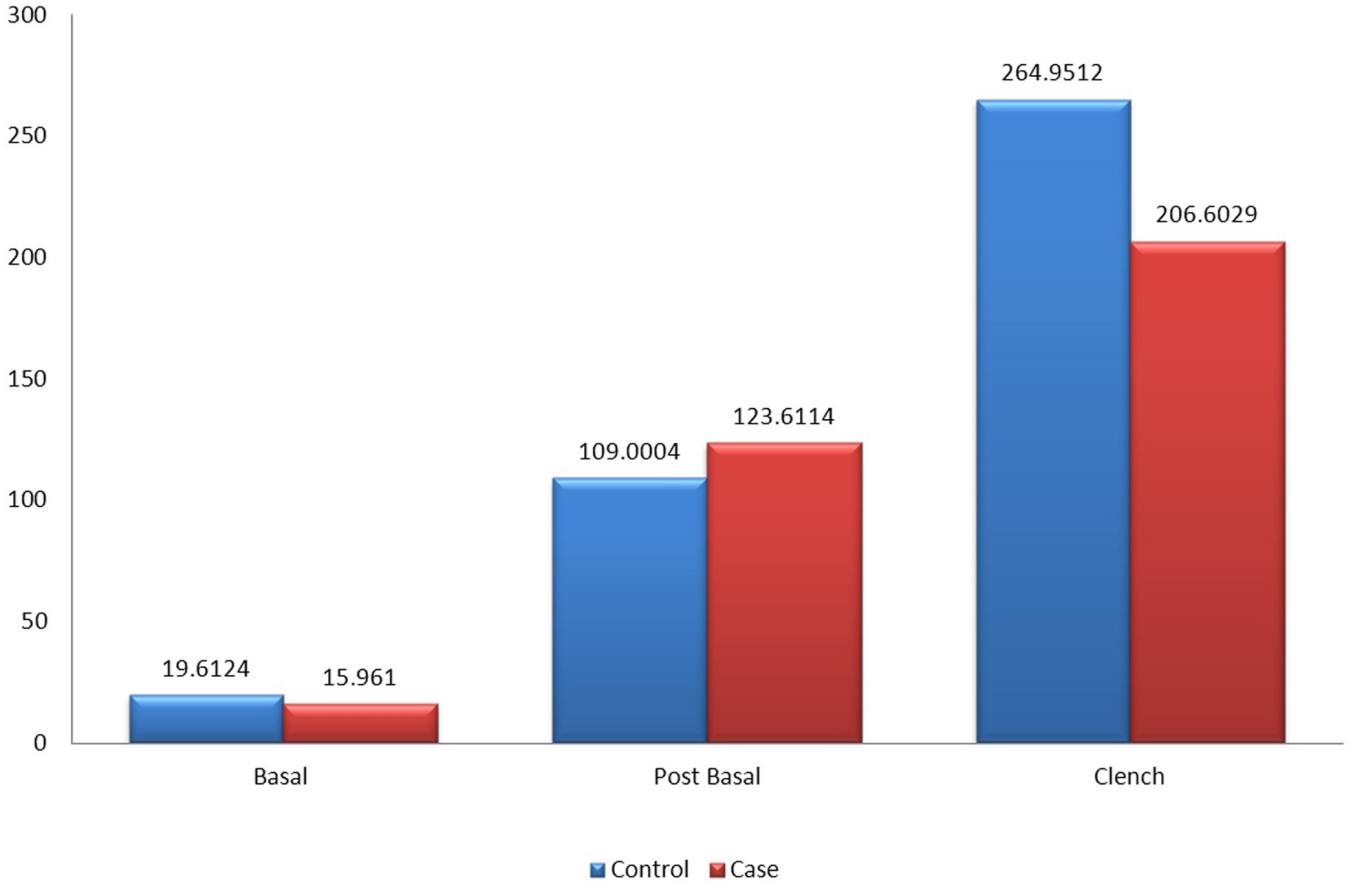
Mean amplitude of the right temporalis muscle between control and patient.

Fig 13 shows that the mean amplitude of the right temporalis muscles between patients and the control group during basal stage (resting), post chew resting stage, post clenching basal stage (resting), left chewing stage, right chewing stage and during clenching stage were not significant.

**Fig 14:**
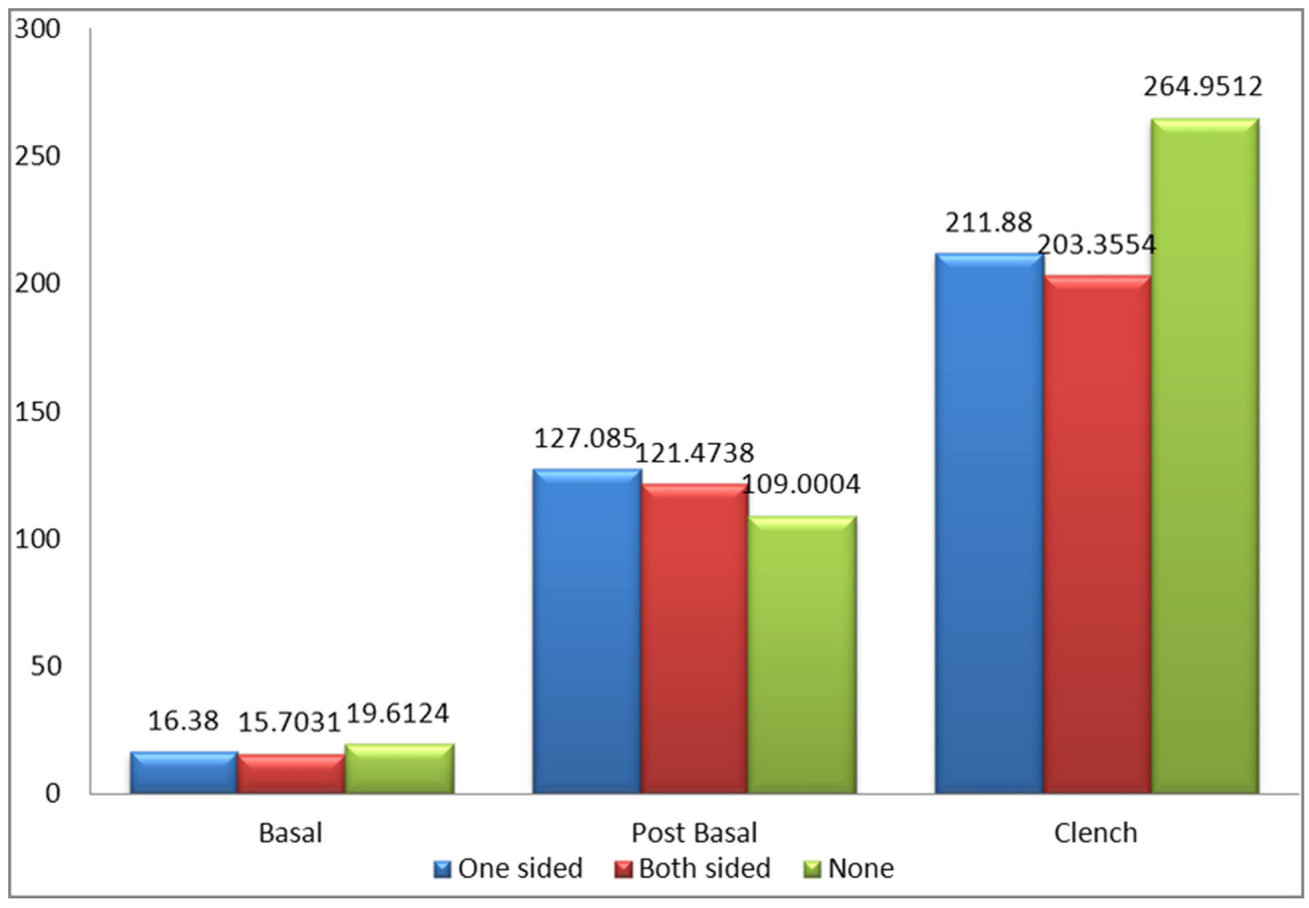
Mean amplitude of the right temporalis muscle between control and patient based on the location of implant.

Fig 14 shows that the mean amplitude of the right temporalis muscles between patients with one sided implants, patients with two sided implants and the control group without implants during basal stage (resting), post chew resting stage, post clenching basal stage (resting), left chewing stage, right chewing stage and during clenching stage were not significant as well.

## Discussion

Demographic data retrieved from the subjects showed that most of the patients who participated in this study were females, 66.6% (14) while the males made up 33.3% (7) of the patients. This is in contrast to a study done by Sulieman et al. in which 74.1% (281) of the respondents were males and 25.9% (98) were females [7]. The age of the patients ranges from 31–65 years old. When classified according to age groups, the highest percentage of patients falls in the range of age 41–50. This is also in contrast to the study above whereby majority of the subjects (51%) were under 30 years of age [7]. Subjects included in this study were 18 years and above as mentioned in the inclusion criteria for participation. Changes in the dentoalveolar complex are of particular importance for the functional/aesthetic outcome of implants, this is because while continuous increase in dentoalveolar height from puberty until middle life generally causes no major changes in the natural occlusion, implants will gradually go into infraocclusion [8]. No significant differences were observed between gender and age groups in all stages of the mean amplitude of the left masseter, right masseter, left temporalis and right temporalis. Hence, it can be said that results obtained from patients in this study are not affected by gender and age factor.

Regarding source of information of which the patients learn about dental implants, most of them were informed by their relatives and friends. Only 3 patients came to know about dental implants from their referring dentists while 5 patients received information from the mass media, particularly the internet as a choice for knowledge about dental implants. This is in contrast to a study done by Satpathy et al. [9] that studied patient awareness and source of information about dental implants where most patients learn about dental implants from their dentists followed by print and electronic media.

Patients were asked regarding the reason for undergoing dental implant placement. A variety of reasons such as restoring lost teeth, to relief discomfort of previous appliance such as crown, bridge or removable dentures, to improve stability of removable dentures, to improve masticatory function and aesthetics were provided in the questionnaire. Majority of the patients who underwent dental implant treatment had the intention to restore lost teeth (due to caries and extraction) while the rest wanted relief from uncomfortable dental appliances (data received was 2 crowns, 2 bridges and 3 removable dentures). This result was in accordance with those concluded by Satpathy et al. [9] where majority of the patients in that study were not content with a removable prosthesis as the treatment in replacing missing teeth. Similarly, in the study by Tepper et al. [10] it showed that a higher percentage of younger and middle aged patients rejected removable dentures. Patients of older age group were more willing to accept functionally poor dentures. This may be because of the fact that they tend to develop compensatory adaptive processes and to some extent unconsciously accept age-related losses of masticatory function [11]. Hence, undergoing dental implant treatment has been the preferred choice of the subjects in this study, especially in their age group where replacing lost teeth with dental implant gave them most satisfaction.

Regarding discomfort related to all phases of treatment, 18 patients denied of any discomfort from the implant procedure taking a long time while only 3 of them felt discomfort due to the procedure taking a long time, of which only 1 patient commented having to wait for a long time before crown placement. According to a study by Spin-Neto et al. [12] that aims to evaluate postoperative discomfort in relation to pain, bleeding, and swelling, together with treatment time, in patients treated with a single tooth implants, it was reported that no other studies have assessed the difference in treatment time and the patients’ perception of treatment discomfort after implant installation with and without immediate mounting of the tooth crown. However, it showed that immediate restoration of the tooth crown in connection with implant placement logically demands a longer treatment time than when the tooth is mounted at a later occasion which supports the results obtained. However, it opposes the opinion of one patient that commented on the long wait before crown placement. The number of patients who felt traumatized by the dental implant treatment was low as only 1 patient reported that it was a traumatic experience. Swelling that occurred post treatment was reported to be present among one third of patients who participated in this study. Regarding the swelling, 4 of them experienced mild swelling, 2 had moderate swelling while 1 experienced severe swelling, making a total of 7 patients out of 21 who had swelling. Swelling peaked on the day after the surgery and decreased the few following days post operatively for 6 of them who experienced mild and moderate swelling, while the patient who had severe swelling reported that the swelling gradually decreased until the following visits for review. The results were similar to a study by Spin-Neto et al. [12] reporting patients had swelling that peaked on the day of surgery and decreased throughout seven days. This is not in agreement with other studies that mention although pain was not an issue, swelling seems to reach its maximum one or two days postoperatively [13,14,15]. In this study however, the implant location may play a role in the postoperative pain and swelling as all subjects had posterior implants. Previous studies suggest implant placement caused more severe inflammation when the procedure involved the posterior regions of the jaws [14,15,16].

Pain perception is a multifactorial phenomenon in which sensory and emotional experiences are involved; it is influenced greatly by the subject’s expectation of pain associated with a given dental procedure [17]. Therefore, quantification of pain levels associated with different surgical procedures should aid in establishing guidelines for communication with prospective implant patients and providing them with realistic expectations. This, in turn, may reduce their anxiety levels and lead to greater acceptance and lesser trauma to patients. Only 9 out of 21 patients experienced pain throughout treatment and rated pain with a score from 1–10. The mean pain score is 4.0, range of which the patients rated pain was 1–7, with 4 patients rating pain at the range of 1–3 (mild), 3 patients rating pain at the range of 4–6 (moderate), and 2 patients reported a pain score ranged at 7–10 (severe). Other than pain, only one patient experienced discomfort post-operatively, reporting numbness that occurred after the dental implant placement. This is in accordance to a study done by Annibali et al. which reported that the post-operative period was free of complications in most cases. Pain was absent in 64.5% of the subjects, and only 5.8% experienced severe pain [18].

Regarding functional and esthetical satisfaction, majority of the patients were satisfied with the implant during function as it provided better masticatory function while only one of them was not satisfied probably due to the delayed placement of crown post operatively. In agreement with the results obtained is a study by Satpathy et al. [9] that reported 37% of patients found fixed nature of dental implants more advantageous. This finding is also in accordance with the results revealed by clinical studies on implanted patients that patients already fitted with implants perceived no difference in chewing compared with natural teeth [19,20,21]. As for aesthetics, all of the patients were satisfied with the appearance of their dental implants as it was similar in form and structure as their natural dentition. This is in agreement with high satisfaction levels of the aesthetic outcome found in other studies. [21] Hence, the number of patients who felt their implant to have the most natural feeling to it was 18 as oppose to only 3 that felt it was strange at the beginning stages of having the implant. This echoes the findings in a study done by Pjetursson et al. whereby most of the respondents got used to the dental implant either immediately or soon after the procedure while the rest needed 2–3 months for the implant to feel like natural teeth [19]. Implant stability was rated by most as good while only one reported moderate stability. The number of patients who had a single visit implant placement was 16 while the rest had multiple visits to the dentist, with one of them having a total of 4 visit post operatively. The reason could be for crown placement or review of the dental implant.

From clinical examination of 55 implants found in 21 patients, majority of them had 2 posterior dental implants, the least being 1 implant and the most recorded was 6 implants in 1 patient. All subjects examined exhibited successful dental implants with no pain, tenderness upon function, no mobility and no exudates. None of the implants were in a compromised state or had poor prognosis. However, 4 patients reported that although they were satisfied with the function and stability of the implant, they experience food impaction between the implant and the adjacent tooth due to insufficient contact point. This could have caused some discomfort and more meticulous care of the implant.

Surface electromyography results were not significant except for the left masseter muscle during clenching phase. When compared between patients with one sided, two sided and control patients without implants, the results were also not significant except for mean amplitude of left masseter muscle during clenching phase when compared between both groups of patients and the control group. Patients who have experienced loss of tooth or multiple teeth will usually have compromised masticatory function and efficiency, which could subject the muscles involved in mastication and the TMJ to undergo adaptive changes that include some form of muscle hypertrophy due to hyper contractility of the muscles and arthropathy of TMJ respectively. Although the insertion of dental implants will help restore normal function, reversal of the adaptive changes requires a long period of time. As reported in a study done by Pasero et al. [22], there was a significant decrease in maximal clenching activity, demonstrated through EMG, in patients with different severities of arthropathy. In another study done by Garret N et al. [23] to assess chewing efficiency after improvements of poorly fitting dentures or insertion of new dentures, it was reported that improvements done to restore occlusion contributed to a reduction in masseter muscle effort albeit the improvement in masticatory function as validated by EMG results.

## Conclusion

The present results showed that,

- patients were satisfied with their implants, which showed successful clinical criterias and no significant difference in EMG results.
- patients who were satisfied with their posterior implants had successful clinical criterias.
- there was no significant difference in sEMG results between patients with posterior implant and control group.
- patients who were satisfied with their posterior implants had successful clinical criterias and no significant difference in sEMG results when compared between the control group.
