# Correction: Dental Implants - Perceiving Patients' Satisfaction in Relation to Clinical and Electromyography Study on Implant Patients

**DOI:** 10.1371/journal.pone.0143902

**Published:** 2015-11-23

**Authors:** Mohammad Khursheed Alam, Shaifulizan Abdul Rahman, Rehana Basri, Tiffany Tang Sing Yi, Justin Wong Si-Jie, Soumendra Saha

The second author’s name is spelled incorrectly. The correct name is: Shaifulizan Abdul Rahman. The correct citation is: Alam MK, Rahman SA, Basri R, Sing Yi TT, Si-Jie JW, Saha S (2015) Dental Implants–Perceiving Patients’ Satisfaction in Relation to Clinical and Electromyography Study on Implant Patients. PLoS ONE 10(10): e0140438. doi:10.1371/journal.pone.0140438
